# Targeting cancer lactate metabolism with synergistic combinations of synthetic catalysts and monocarboxylate transporter inhibitors

**DOI:** 10.1007/s00775-023-01994-3

**Published:** 2023-03-08

**Authors:** Hannah E. Bridgewater, Elizabeth M. Bolitho, Isolda Romero-Canelón, Peter J. Sadler, James P. C. Coverdale

**Affiliations:** 1 Department of Chemistry, University of Warwick, Gibbet Hill Road, Coventry, CV4 7AL UK; 2 Centre of Exercise, Sport and Life Science, Faculty of Health and Life Sciences, Coventry University, Coventry, CV1 5FB UK; 3 School of Pharmacy, Institute of Clinical Sciences, University of Birmingham, Edgbaston, Birmingham, B15 2TT UK

**Keywords:** Catalysis, Osmium, Cancer, Lactate, Redox, Organometallic, AZD3965

## Abstract

Synthetic anticancer catalysts offer potential for low-dose therapy and the targeting of biochemical pathways in novel ways. Chiral organo-osmium complexes, for example, can catalyse the asymmetric transfer hydrogenation of pyruvate, a key substrate for energy generation, in cells. However, small-molecule synthetic catalysts are readily poisoned and there is a need to optimise their activity before this occurs, or to avoid this occurring. We show that the activity of the synthetic organometallic redox catalyst [Os(*p*-cymene)(TsDPEN)] (**1**), which can reduce pyruvate to un-natural d-lactate in MCF7 breast cancer cells using formate as a hydride source, is significantly increased in combination with the monocarboxylate transporter (MCT) inhibitor AZD3965. AZD3965, a drug currently in clinical trials, also significantly lowers the intracellular level of glutathione and increases mitochondrial metabolism. These synergistic mechanisms of reductive stress induced by **1**, blockade of lactate efflux, and oxidative stress induced by AZD3965 provide a strategy for low-dose combination therapy with novel mechanisms of action.

**Graphical abstract:**

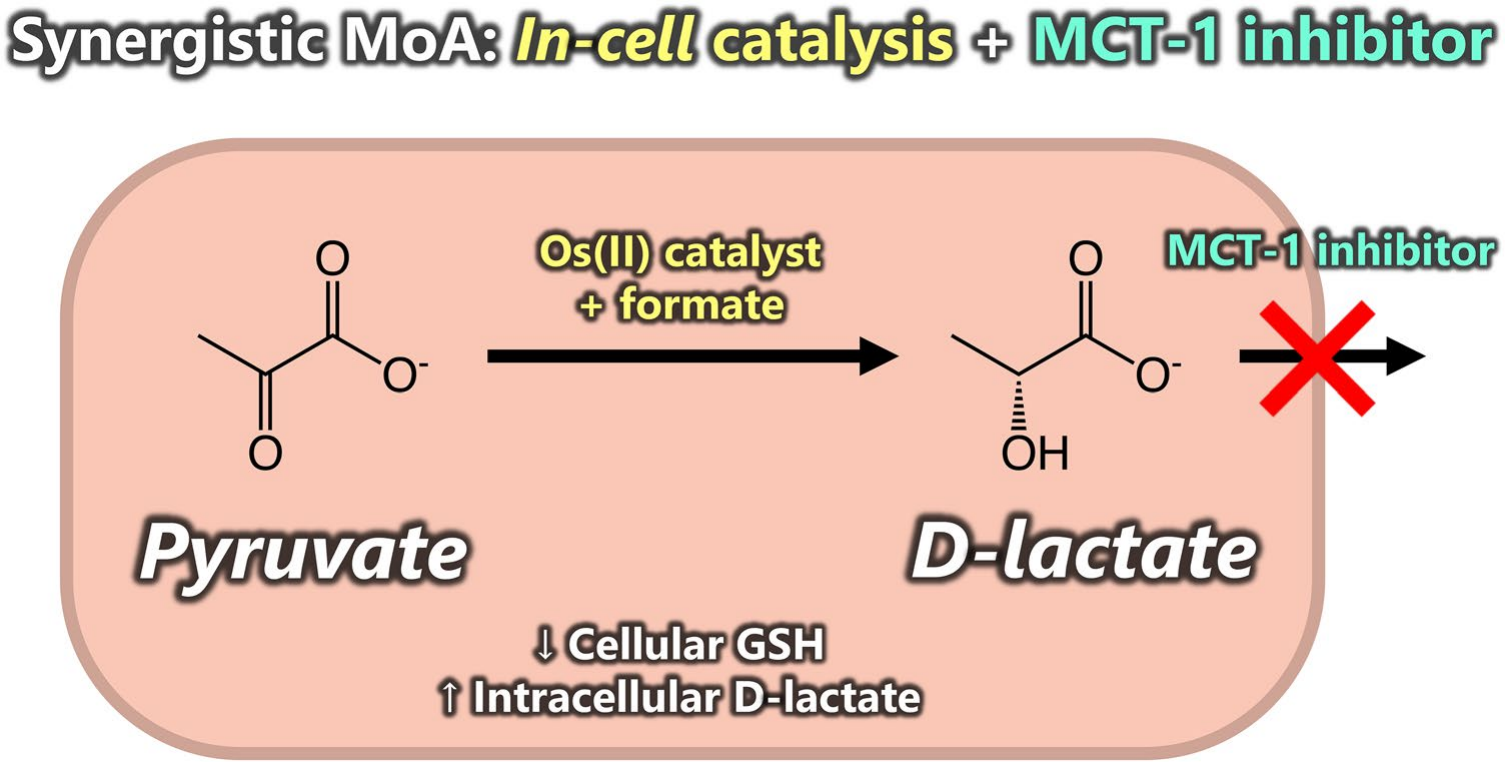

**Supplementary Information:**

The online version contains supplementary material available at 10.1007/s00775-023-01994-3.

## Introduction

In recent years, there has been considerable progress in the design of small-molecule synthetic compounds which are active as catalysts in living cells [1–9]. Synthetic superoxide dismutase mimics, based on metal complexes, nanomaterials or protein–metal conjugates, have been extensively explored [10]. Ir(III) pyridinecarboxamidate complexes can catalyse the in-cell reduction of aldehydes to alcohols, using NADH as a source of hydride [11], whilst Ru(II) and Rh(III) arene sulfonamidoethylenediamine and Schiff base catalysts have been developed which allow the in-cell reduction of NAD^+^ [12–15]. Inorganic catalysts, therefore, offer potential as novel drugs with new mechanisms of action. However, there are significant challenges to overcome on account of the large number of competing nucleophiles in cells, possible poisons for synthetic catalysts. Unlike metalloenzymes, catalytic metal sites in small molecules usually have exposed substrate binding sites. Nonetheless, we have shown that with appropriate choice of a relatively inert third-row transition metal ion osmium(II), a π-bound arene, a substituted chelated diamine ligand which stabilises the active 16-electron coordinatively unsaturated catalyst, and chiral centres on the diamine backbone and the metal, then in-cell enantioselective transfer hydrogenation catalysis can be achieved, using a non-toxic concentration of sodium formate (a naturally occurring metabolite in human plasma) as a source of hydride [16]. These catalysts can carry out the enantioselective reduction of pyruvate, the end product of glycolysis, to un-natural d-lactate in cells [17, 18].

**Fig. 1:**
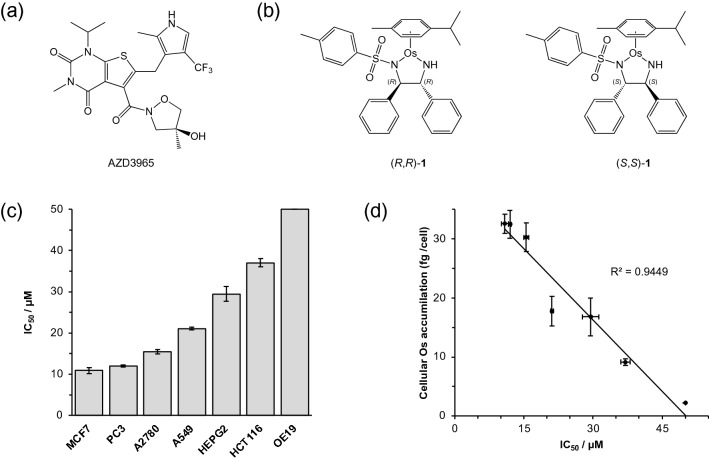
**a** Structure of the MCT-1 inhibitor, AZD3965. **b** Structure of Os asymmetric transfer hydrogenation catalyst (*R*,*R*)-**1** and its enantiomer, (*S*,*S*)-**1**. **c** Antiproliferative activity (IC_50_/μM) screening of (*R*,*R*)-**1** towards seven human cancer cell lines: A2780 (ovarian carcinoma), A549 (lung adenocarcinoma), HCT116 (colorectal carcinoma), HEPG2 (hepatocellular carcinoma), OE19 (oesophageal carcinoma), MCF7 (breast adenocarcinoma), and PC3 (prostate carcinoma); determined using the sulforhodamine B cell viability assay (24 h exposure, 72 h recovery time). Error bars represent ± 1 S.D. from the mean. No error bars are shown for antiproliferative activity in OE19 cells (IC_50_ > 50 μM). **d** Linear correlation between antiproliferative activity (IC_50_/μM) and cellular osmium accumulation (*R*^2^ = 0.9449). Full numerical and statistical data can be found in Supplementary Information Tables S1–S3

Still, the pathophysiology of d-lactate remains relatively poorly understood. The normal plasma concentration of d-lactate (*ca*. 10 μM) is typically two orders of magnitude lower than that of l-lactate, and d-lactate appears to be eliminated at approximately half the rate of l-lactate, being mainly oxidised to pyruvate prior to excretion [19]. Lactate metabolism is considered to be an attractive target for new anticancer therapies [20–22]. Dysregulation of monocarboxylate transporters (MCTs) and lactate dehydrogenase isoforms are thought to contribute to the Warburg effect observed in cancer cells [23]. In particular, monocarboxylate transporter 1 (MCT-1) plays an important role in lactate signalling, facilitating both cellular influx and efflux [24]. MCT-1 is upregulated in many cancer cell lines, and a clear correlation has been identified between MCT-1 overexpression, cancer tumorigenicity, and poor clinical prognosis [25, 26]. As such, the development of selective MCT-1 inhibitors has gained increasing attention [25–27]. MCT-1 inhibition leads to the accumulation of intracellular lactate, decreased tumour cell growth, a slower rate of glycolysis, and lower intracellular concentrations of ATP, NADPH, and the tripeptide antioxidant glutathione (GSH) [28]. It has also been suggested that MCT-1 inhibition acts to block pyruvate export [29]. The inhibitor AZD3965 (Fig. 1a) entered Phase I/II clinical trials in the United Kingdom in 2013 [30, 31], and has ~ tenfold selectivity for MCT-1 (*K*_i_ = 1.6 nmol L^−1^) over MCT-2 (*K*_i_ = 20 nmol L^−1^). In addition to lactate transport inhibition, this MCT-1 inhibitor increases the concentration of TCA cycle metabolites [32], and decreases tumour choline levels [33]. Though MCT-1 is also known to transport formate (the reducing agent required for in-cell catalytic reduction), the K_m_ for formate transport by MCT-1 is up to two orders of magnitude greater than that of pyruvate or lactate [34]. A non-toxic concentration of MCT-1 inhibitor may, therefore, block pyruvate and/or lactate export, whilst still allowing import of formate as hydride source.

In this work, we combine both approaches and have investigated a synergistic combination of a catalytic organometallic anticancer transfer hydrogenation complex, using formate as a source of hydride, with an MCT inhibitor. By blocking lactate efflux, the overall potency of the metallodrug can be enhanced and this combination can act to perturb lactate metabolism via the generation of un-natural d-lactate. We find that the combination of an MCT-1 inhibitor with a catalytic metallodrug not only increases the in-cell catalytic turnover number (TON) for the reduction of pyruvate to d-lactate, but also enhances the anticancer potency of the catalyst by lowering the cytosolic concentration of GSH.

## Results and discussion

### Activity of synthetic catalyst in MCF7 breast cancer cells

The two enantiomers of a 16-electron organo-osmium catalyst, (*R*,*R*)-**1** and (*S*,*S*)-**1**, [Os(*p*-cymene)(TsDPEN)] are known to catalyse the asymmetric transfer hydrogenation (ATH) of ketones to afford optically pure alcohols in the presence of a suitable hydride donor in living cells (Fig. 1b). These Os(II) catalysts were synthesised and fully characterised as previously described. They are highly stable in DMSO (used to enhance solubility) and in culture medium containing 10% foetal calf serum over a 24 h period [17]. Using complementary inductively coupled plasma-mass spectrometry (ICP-MS) and X-ray fluorescence (XRF) elemental mapping, Os TsDPEN catalysts have been shown to be delivered to cells intact to carry out intracellular catalysis reactions [35]. The diamine ligand is eventually displaced by endogenous thiols and translocated to the nucleus, whilst the Os fragment is exported from cells; however, cellular accumulation studies (a measure of the influx/efflux dynamic equilibrium) demonstrated that Os remains present in cells after 24 h [35]. Both of these catalytic enantiomers were screened for activity towards seven human cancer cell lines (A2780, ovarian carcinoma; A549, lung adenocarcinoma; HCT116, colorectal carcinoma; HEPG2, hepatocellular carcinoma; OE19, oesophageal carcinoma; MCF7, breast adenocarcinoma; and PC3, prostate adenocarcinoma, Fig. 1c) to identify the cell line in which the highest cytotoxic potency (lowest IC_50_ concentration) and cellular accumulation (femtograms of osmium per cell) could be achieved (Supplementary Information Tables S1–S3).

Antiproliferative activities (IC_50_ concentrations) strongly correlated (*R*^2^ = 0.9449) with cellular osmium accumulation (Fig. 1d) and were found to be similar for the two catalytic enantiomers (Supplementary Information Table S1). We have previously shown that Os accumulation in ovarian cancer cells involves contributions from both passive diffusion and active transport mechanisms [17]. It is noteworthy that accumulation measurements determined here for various cancer cell lines using equipotent concentrations of catalyst** 1** (Supplementary Information Table 2), display an inverse correlation (i.e. cells treated with the lowest concentration of **1** accumulated the most intracellular Os), though it is unclear whether this phenomenon is due to active transportation mechanism (since passive diffusion would typically increase accumulation with respect to concentration) or passive diffusion (depending on the hydrophobicity of the compound and its ability to cross cell membranes passively). The highest potency (IC_50_ = 11 ± 1 μM) and greatest accumulation (33 ± 2 fg Os cell^−1^) were observed for MCF7 breast cancer cells. Importantly, MCF7 cells are known to overexpress MCT-1 [23], and thus were highly suitable for studies of the enhancement of in-cell lactate accumulation by the synthetic catalyst in combination with an MCT-1 inhibitor. As such, MCF7 cells were chosen for detailed studies of the effect of the synthetic catalyst since these cells were likely to contain the highest level of intracellular catalyst.

**Fig. 2:**
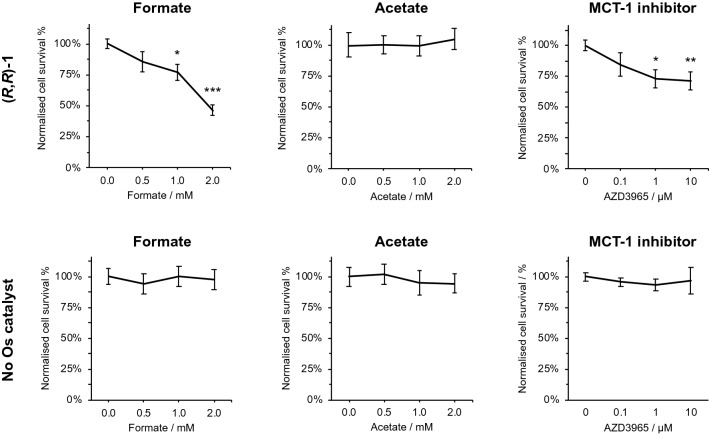
Antiproliferative activity modulation of catalyst **1** in MCF7 breast cancer cells (only *R*,*R*-**1** shown for clarity, 5.5 μM, 0.5 × IC_50_ concentration) by sodium formate, 0–2 mM; or sodium acetate, 0–2 mM; or MCT-1 inhibitor AZD3965, 0–10 μM, alone. Modulation experiments were repeated in the absence of catalyst **1**. Cell viability was determined using the sulforhodamine B assay. Sodium formate can act as a suitable hydride donor to facilitate in-cell reduction, significantly decreasing cell survival relative to the formate-free control. Sodium acetate cannot act as a hydride donor for in-cell reduction catalysis. The MCT-1 inhibitor (AZD3965) increases the potency of catalyst **1** relative to the inhibitor-free control. Non-toxic concentrations of formate, acetate and AZD3965 do not affect cell survival in the absence of catalyst **1**. Full numerical and statistical data for activity modulation experiments using (*R*,*R*)-**1** and (*S*,*S*)-**1** can be found in Supplementary Information Tables S4–S5. Error bars represent ± 1 S.D. from the mean. Statistical significances were determined using a two-tailed *t* test assuming unequal sample variances (*p < 0.05, **p < 0.01, ***p < 0.001)

**Fig. 3:**
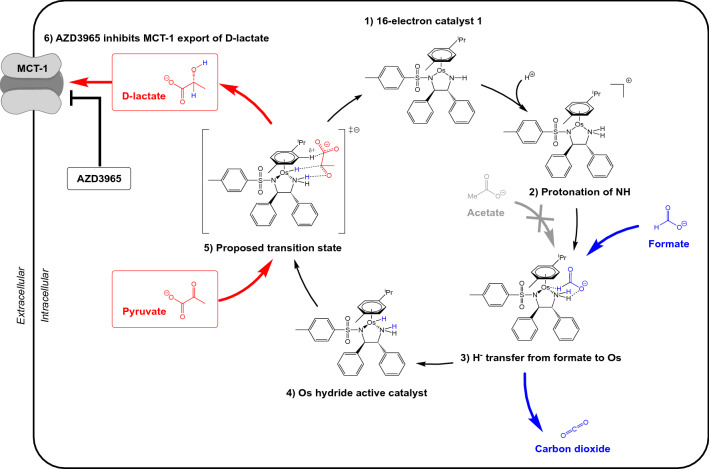
Schematic representation of the in-cell asymmetric transfer hydrogenation of pyruvate to d-lactate (red) by synthetic catalyst (*R*,*R*)-**1**, using formate (blue) as a source of hydride (H^−^). (1) Catalyst (*R*,*R*)-**1** is a coordinatively unsaturated 16-electron complex. (2) Catalyst (*R*,*R*)-**1** protonates to form a cationic intermediate. (3) Formate coordinates via a pseudo-6-coordinate transition state [36]. Acetate cannot act as a suitable hydride donor. (4) An Os-hydride complex is formed, which has previously been observed using ^1^H-NMR [38]. (5) The active hydride catalyst transfers hydride (H^−^) and a proton (H^+^) to the substrate, pyruvate. The proposed transition state for this transfer is shown [17], in which a favourable interaction between the catalyst arene and substrate carboxylate controls substrate orientation, resulting in the observed enantioselectivity for the reduction to d-lactate. Catalyst (*R*,*R*)-**1** is regenerated after hydride/proton transfer to the substrate. 6) AZD3965 is a potent inhibitor of the monocarboxylate transporter MCT-1, which prevents export of d-lactate from the intracellular environment

Co-administration of a non-toxic concentration of sodium formate (0–2 mM), as a reducing agent and hydride donor, alongside catalyst **1** resulted in a highly significant enhancement of the cytotoxic potency of catalyst **1** by up to 60% towards MCF7 breast cancer cells (*p* < 0.001, Fig. 2 and Supplementary Information Table S4). This catalytic mechanism of action was previously observed using catalyst **1** and formate in A2780 ovarian cancer cells, A549 lung cancer cells and PC3 prostate cancer cells, and is conserved in MCF7 breast cancer cells [17, 35]. The mechanism, shown in Fig. 3, involves hydride transfer from formate to Os(II), followed by enantioselective hydride transfer to pyruvate. The *S,S* or *R,R* chiral centres reside on the carbons in the diamine backbone, although there is also chirality associated with the orientation of the *p*-cymene arene ligand, the osmium centre itself, and the pucker of NCH_2_CH_2_N fragment of the chelate ring [36].

Interestingly, the modulation of antiproliferative activity by co-administration of sodium formate was statistically similar between either enantiomer of the catalyst: (*R*,*R*)-**1** or (*S*,*S*)-**1** (Supplementary Information Table S4). To confirm the transfer hydrogenation (TH) catalytic mechanism, activity modulation experiments using acetate in place of formate were performed (Fig. 2). For either enantiomer of catalyst **1**, cell survival was unaffected (*p* > 0.05) by the presence of acetate. Acetate cannot act as a hydride donor, thus the specific role of formate as a hydride donor for intracellular reduction was confirmed. This observation is consistent with previous reports which highlight the role of acetate in intracellular catalysis [13]. To further support evidence of intracellular catalysis, cellular Os accumulation studies demonstrated that increased catalyst potency was not the result of increased osmium accumulation somehow caused by formate, since total cellular osmium content was constant irrespective of formate co-administration (Supplementary Information Table S3).

### Combination of a synthetic catalyst with an MCT-1 inhibitor enhances d-lactate generation

**Fig. 4:**
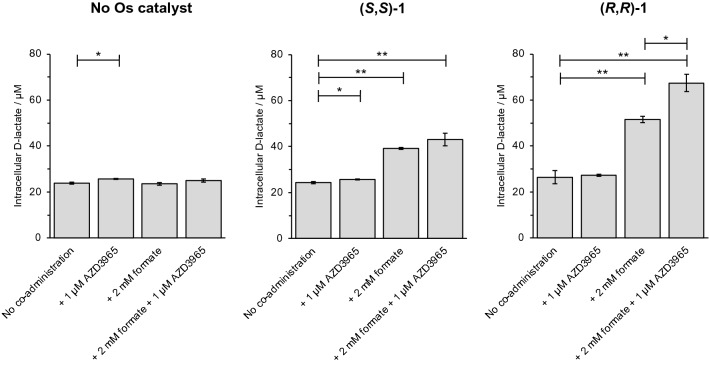
Quantification of intracellular d-lactate (μM) in MCF7 breast cancer cells. Catalyst (*R*,*R*)-**1** or (*S*,*S*)-**1** (11 μM, 1 × IC_50_ concentration) was administered in combination with sodium formate (2 mM) and the MCT-1 inhibitor AZD3965 (1 μM) for 24 h. In combination, catalyst **1**, formate and AZD3965 significantly increase the concentration of intracellular d-lactate. Full numerical and statistical data can be found in Supplementary Information Tables S6. Error bars represent ± 1 S.D. from the mean. Statistical significances were determined using a two-tailed *t* test assuming unequal sample variances (*p < 0.05, **p < 0.01)

Modulation of lactate metabolism in MCF7 breast cancer cells was investigated by quantifying intracellular d-lactate concentrations (Fig. 4). In cells, lactate dehydrogenase primarily converts cytosolic pyruvate to l-lactate, though it is known that cells may also contain a small proportion of d-lactate, a product of the methylglyoxal pathway [37]. Using catalyst (*R,R*)-**1** which can reduce pyruvate to d-lactate enantioselectively in vitro [17], with sodium formate, the cellular d-lactate concentration significantly increased (*p* < 0.01). Reductive enantioselectivity was retained in MCF7 cells, with higher intracellular d-lactate levels generated in the presence of (*R*,*R*)-**1** compared to (*S*,*S*)-**1** (d-lactate: 52 ± 1 µM and 39.2 ± 0.4 µM, respectively). These experiments were carried out using pyruvate-free cell culture medium, and, therefore, it is highly likely that this transformation occurs intracellularly, and not in the extracellular medium in which there is no reagent or reaction that would account for this transformation.

Next, MCF7 cells were co-treated with **1** (catalyst), formate (hydride source for catalytic reduction), and the MCT-1 inhibitor AZD3965, (inhibits efflux of the catalytic reduction product, d-lactate). Importantly, inhibition of MCT-1 can be expected to affect the uptake of acetate, but not formate, since although the latter is also a monocarboxylate, it is transported by Slc26a5 and Slc26a6) [39]. This combination treatment enhanced the cellular level of d-lactate by ca. 2.8-fold for (*R*,*R*)-**1** (67 ± 4 µM) relative to both untreated cells (23.9 ± 0.4 µM) or levels determined in the absence of AZD3965 (52 ± 1 µM). When administered in the absence of catalyst **1** or formate, the MCT-1 inhibitor increased intracellular d-lactate levels only slightly compared to untreated cells (25.7 ± 0.3 µM compared to 23.9 ± 0.4 µM), attributable to reduced efflux of endogenous d-lactate (Fig. 4). Neither enantiomer of catalyst **1** alone increased d-lactate levels, confirming the vital role of the hydride donor in the reductive transformation. Similarly, co-administration of either enantiomer of catalyst **1** with AZD3965 in the absence of formate (the co-factor for catalytic reduction) did not significantly increase the intracellular d-lactate concentration. Though it is likely that co-administration of the MCT-1 inhibitor inhibits efflux of d-lactate, it may also increase the availability of the substrate, pyruvate.

The enantioselectivity observed in the generation of d-lactate is attributable to the chirality of the diamine ligand. Whilst (*R*,*R*) and (*S*,*S*) enantiomers display equal cytotoxic potency in the absence of formate (i.e. without initiation of catalysis), activation of catalysis by co-administration of sodium formate allows (*R*,*R*)-configured catalyst **1** to generate d-lactate preferentially. The (*S*,*S*)-enantiomer has previously been shown to produce l-lactate in an in vitro chemical model system [17]. The accurate quantification of micromolar changes in l-lactate concentration in a cellular environment containing millimolar concentrations of l-lactate was not feasible in view of the experimental errors associated with the methodology used.

Direct measurement of an in-cell catalytic turnover number (TON) requires an assumption to be made about the concentration of the active in-cell catalyst. We have assumed the average volume of an MCF7 cell of *ca*. 1760 μm^3^, and that 5% of the catalyst remains available in its active form due to partitioning amongst cellular organelles and interactions with proteins and other biomolecules [17, 40]. Given Os accumulation, measured in MCF7 cells (Supplementary Table S3) and d-lactate generated (Supplementary Table S6) as input parameters, the TON in cells treated with catalyst **1** and sodium formate (no MCT-1 inhibitor) for 24 h is *ca*. 19. In comparison, we previously determined a TON of *ca*. 13 (24 h) in A2780 human ovarian cancer cells under similar experimental conditions [17]. In the presence of AZD3965, the catalyst turnover number in MCF7 cells shows an apparent *ca*. 50% increase, to a calculated TON of *ca*. 30 (24 h). This increase can be attributed to inhibition of d-lactate efflux. In the absence of AZD3965, some catalytically generated d-lactate would have been exported from the intracellular environment, and the true catalytic turnover number is likely to be underestimated. It is likely that this organometallic catalyst is multi-targeting and may catalyse several intracellular transformations as previously demonstrated for structurally similar Ru complexes (e.g. the reduction of NAD^+^ to NADH). In identifying only the generation of d-lactate in this study, TON calculations may significantly underestimate the true performance of this catalyst in cells. Furthermore, we cannot rule out direct (non-catalytic) action of the metal complexes on subcellular organelles, such as mitochondria or the ER, since **1** displayed modest potency in the absence of formate. We have previously shown that millimolar concentrations of lactate are not cytotoxic to cancer cells in culture [17], and as such the catalytic generation of lactate alone cannot satisfactorily explain the enhanced potency of catalyst **1** towards cancer cells.

### Depletion of intracellular glutathione by AZD3965

The concentration of intracellular d-lactate was not increased by (*R*,*R*)-**1** and AZD3965 in the absence of an added hydride donor. Direct enhancement of catalyst (*R*,*R*)-**1** potency by co-administration of AZD3965 alone was inconclusive (11 ± 1 μM in the absence of AZD3965, 8 ± 2 μM in the presence of 1 μM AZD3965, Fig. 2 and Supplementary Information Tables S5 and S7). However, AZD3965 is known to deplete the level of intracellular glutathione [28], the thiol-containing tripeptide γ-L-Glu-L-Cys-Gly (GSH), an antioxidant known to coordinate to transition metal drugs [28, 41, 42]. Though similar Ru-thiolato adducts may be active and contribute to a lack of cross-resistance with platinum drugs [43], the vacant Os coordination site on coordinatively unsaturated catalyst **1** is essential to allow hydride binding in the ATH catalysis mechanism [36, 38]. Therefore, the activity of AZD3965 in decreasing intracellular GSH levels in MCF7 cells may contribute to reducing the interaction of GSH and consequent poisoning of an intracellular catalyst. Alternatively, given complex **1** has been shown to induce the generation of reactive oxygen species (ROS) in vitro and in vivo [44], depletion of intracellular GSH by AZD3965 may leave the cell inherently more vulnerable to such a redox-targeted therapy.

**Fig. 5:**
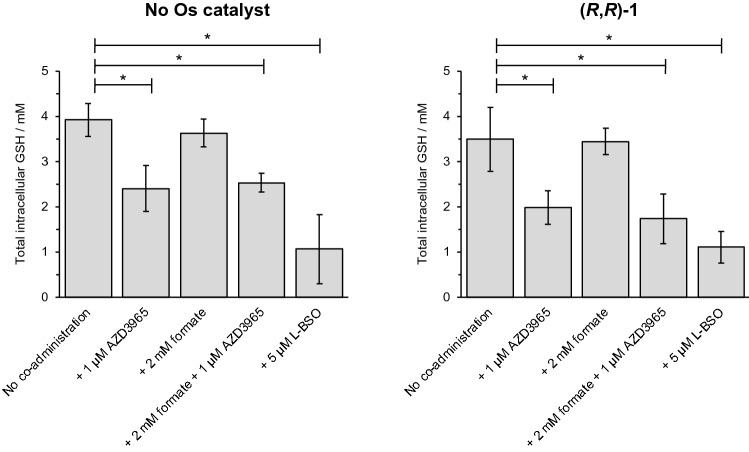
Determination of total intracellular glutathione (mM). MCF7 breast cancer cells were treated with (*R*,*R*)-**1** in the presence/absence of sodium formate (2 mM), MCT-1 inhibitor AZD3965 (1 μM), or L-BSO (5 μM) for 24 h. Glutathione (GSH) levels were not significantly affected by the presence of catalyst **1** or formate, however AZD3965 significantly depleted total cellular GSH. L-Buthionine sulfoximine (L-BSO), a known inhibitor of GSH synthesis [45, 46], depleted GSH levels slightly more than AZD3965. Full numerical and statistical data can be found in Supplementary Information Tables S9–S10. Error bars represent ± 1 S.D. from the mean. Statistical significances were determined using a two-tailed *t* test assuming unequal sample variances (*p < 0.05).

Intracellular GSH levels were measured in MCF7 cells treated with a combination of catalyst (*R*,*R*)-**1**, formate and/or AZD3965 (Fig. 5 and Supplementary Information Table S9). In the presence of catalyst **1** and/or formate, total cellular GSH was not affected relative to the untreated control. In contrast, total cellular GSH was significantly depleted by up to *ca*. 50% (*p* < 0.05) under all experimental conditions which included the presence of the MCT-1 inhibitor. To explore whether the potency of catalyst **1** can be modulated by changes in intracellular GSH levels, catalyst **1** was co-administered to MCF7 cells alongside L-buthionine sulfoximine (L-BSO), a known inhibitor of GSH synthesis [45, 46]. Considering the inherent experimental error, it is unclear whether depletion of intracellular GSH by L-BSO (*ca*. 70% GSH depletion, Fig. 5 and Supplementary Information Tables S8–10) had a meaningful impact on the potency of catalyst **1**. However, previous mass spectrometric studies have demonstrated that catalyst **1** can be deactivated by thiols (cysteine) in chemical model systems at both neutral and acidic pH [35]. Alternatively, GSH depletion by AZD3965 may indirectly affect the potency of catalyst **1** in combination with AZD3965. Decreased intracellular GSH, caused by MCT-1 inhibition, has been shown to increase oxidative stress and cause mitochondrial damage [28, 47]. Since the mechanism of action of catalyst **1** has been shown to involve redox-targeting and generation of reactive oxygen species in vivo [44], AZD3965 and catalyst **1** may independently act to increase cellular oxidative stress. Nonetheless, AZD3965 appears to be dual-acting in its enhancement of the activity of catalyst **1** towards MCF7 breast cancer cells: (1) by increasing cellular d-lactate generated by the in-cell reduction of pyruvate, and (2) by depleting total cellular GSH.

## Conclusions

Lactate metabolism is an attractive target for new cancer therapies. Such an approach has been shown to supress breast tumour growth in vivo [48]. Combinatorial approaches for the treatment of cancer are clinically well established and have been extensively reviewed [49]. Combinations which act synergistically are particularly promising (for example the combination of a caspase inhibitor and an apoptosis inducer) and aim to increase clinical efficacy and potentially reduce the likelihood of drug resistance development. Here, a potent MCT-1 inhibitor offers dual enhancement of a novel catalytic metallodrug. The concentration of intracellular d-lactate, generated catalytically by the catalyst in the presence of formate, is significantly enhanced in the presence of AZD3965, whilst the depletion of intracellular GSH by AZD3965 further increases redox stress in cancer cells, and may reduce catalyst deactivation by GSH adduct formation. It remains unclear whether AZD3965 acts solely to disrupt lactate metabolism, and/or also influences substrate (pyruvate) or co-factor (formate) availability. The catalytic reduction of pyruvate to d-lactate is likely to be only one of many intracellular targets or pathways affected by Os catalyst **1**, since pyruvate and/or lactate are unlikely to be the sole cause of cell death (we have previously demonstrated that millimolar levels of these metabolites are non-toxic to cells) [17]. Future metabolomic, proteomic and genomic studies are likely to provide a deeper understanding of the mechanism of action, and how these processes influence the wider cellular environment. Nonetheless, our findings are an important step in showing how catalytic drugs might eventually be applied as part of a drug combination treatment in the clinic.

Our work provides evidence for the catalytic mechanism which generates d-lactate (an un-natural metabolite which would otherwise occur only at very low levels) by catalyst **1** in cancer cells, and enhancement of its intracellular concentration using AZD3965. Future investigations employing isotopically labelled formate may help to elucidate the cellular import, export, and localisation of this hydride donor (formate), substrate (pyruvate), and reduction product (d-lactate). It will also be interesting to investigate whether there is selective enhancement of potency for this new combination treatment towards cancer cells versus normal cells (as appeared to be the case in comparisons of the activity of catalyst **1** and formate in cancerous and non-cancerous ovarian cells) [17], and to extend the work to in vivo experiments.

## Supplementary Information

Below is the link to the electronic supplementary material.

Supplementary file1 (PDF 296 KB)

## Data Availability

The data that support the findings of this study are available from the corresponding author upon reasonable request.
