# Supplementary material for: Targeting cancer lactate metabolism with synergistic combinations of synthetic catalysts and monocarboxylate transporter inhibitors
Source: J Biol Inorg Chem. 2023 Mar 8;28(3):345–53. doi: 10.1007/s00775-023-01994-3 (PMC10036267; doi:10.1007/s00775-023-01994-3)
Supplement: Supplementary file 1 — Supplementary file1 (PDF 296 KB) [file 775_2023_1994_MOESM1_ESM.pdf]

Targeting cancer lactate metabolism with synergistic combinations of synthetic catalysts and monocarboxylate transporter inhibitors

Hannah E. Bridgewater,[1,2] Elizabeth M. Bolitho,[1] Isolda Romero-Canelón,[1,3] Peter J. Sadler,[1] and James P. C. Coverdale*[1,3]

[1] Department of Chemistry, University of Warwick, Gibbet Hill Road, Coventry, CV4 7AL, UK
[2] Faculty of Health and Life Sciences, Coventry University, Coventry, CV1 5FB, UK
[3] School of Pharmacy, Institute of Clinical Sciences, University of Birmingham, Edgbaston, B15 2TT, UK
* Corresponding author: j.p.coverdale@bham.ac.uk

Supporting Information

Table of contents

Materials and chemical synthesis S2

Biological studies S3

Tables **S1-S9** S5

Materials and chemical synthesis

Potassium hydroxide and anhydrous magnesium sulphate were obtained from Fisher Scientific. (*R*,*R*)- and (*S*,*S*)-*N*-tosyl-diphenylethylenediamine were obtained from Arran Chemical Company Ltd (Ireland) as enantiopure materials. Osmium dimer [Os(*p*-cymene)Cl_2]$_2$ was prepared following an established microwave method.[37, 49] Trace metal grade nitric acid was freshly distilled before use. Human cancer cell lines (A2780, A549, HCT116, OE19, MCF7, and PC3) were obtained from the European Collection of Cell Cultures (ECACC) and tested for mycoplasma-free status biannually. AZD3965 (≥95% purity by HPLC) and the D-lactate assay kit were purchased from Cayman Chemical. The intracellular glutathione assay was purchased from Thermo Fisher Scientific. All other solvents, reagents and consumables were purchased from Merck and used as received.

Synthesis of catalyst 1 [Os(*p*-cymene)(*N*-tosyl-diphenylethylenediamine)]. Catalyst **1** was prepared using a previously reported method.[17, 37] To a solution of osmium dimer [Os(*p*-cymene)Cl_2]$_2$ (51.4 mg, 0.07 mmol, 1.0 mol equiv.) and either (*R*,*R*) or (*S*,*S*)-*N*-tosyl-diphenylethylenediamine (51.3 mg, 0.14 mmol, 2.1 mol equiv.) in dichloromethane (25 mL) was added potassium hydroxide pellets (56.1 mg, 1.0 0 mmol, 15 mol equiv.) and water (25 mL). The biphasic reaction was stirred vigorously to agitate the solvent interface and the solution turned from yellow to dark red. The red organic phase was diluted with additional dichloromethane (25 mL), washed with water (2 × 50 mL) and dried over anhydrous magnesium sulphate. The solvent was removed under reduced pressure to afford a dark red amorphous solid, which was recrystallised from dichloromethane/hexane (68.0 mg, 0.100 mol, 75 %). ^{1}H NMR (400 MHz, $CDCl_3$, 25°C, TMS): δ = 7.41 (d, ^3J(H,H)=7.6 Hz, 2H), 7.05-7.20 (m, 10H), 6.82 (d, ^3J(H,H)=8.0 Hz, 2H), 6.80 (br s, 1H; NH), 5.79 (d, ^3J(H,H)=5.6 Hz, 1H; Os-ArH), 5.62 (d, ^3J(H,H)=5.6 Hz, 1H; Os-ArH), 5.52 (d, ^3J(H,H)=5.6 Hz, 1H; Os-ArH), 5.42 (d, ^3J(H,H)=5.6 Hz, 1H; Os-ArH), 4.42 (s, 1H; $CHCHNH_2$), 3.94 (d, ^3J(H,H)=4.3 Hz, 1H; TsNCH), 2.45 (sept, ^3J(H,H)=6.9 Hz, 1H; $CH(CH_3)_2$), 2.23 (s, 3H; CH_3), 2.22 (s, 3H; CH_3), 1.23 (d, ^3J(H,H)=6.9 Hz, 3H; $CH(CH_3)_2$), 1.17 (d, ^3J(H,H)=6.9 Hz, 3H; $CH(CH_3)_2$); 13C NMR (100 MHz, $CDCl_3$, 25°C, TMS) δ = 127.4, 127.0, 126.8, 126.0, 125.9, 125.9, 125.4, 81.7, 76.2, 72.4, 70.7, 70.0, 66.2, 22.5, 22.4, 20.2; UV/Vis: λ_{max} 260, 410 and 478 nm; HRMS (ESI): m/z calculated for $C_{31}H_{35}N_2O_2OsS$ [M+H$^+$]: 691.2028. Found: 691.2031. Elemental analysis for (*R*,*R*)-**1**: (calculated, found for $C_{31}H_{34}N_2O_2OsS$): C (54.05, 53.66), H (4.97, 4.88), N (4.07, 3.95); Elemental analysis for (*S*,*S*)-**1**: (calculated, found for $C_{31}H_{34}N_2O_2OsS$): C (54.05, 53.71), H (4.97, 4.84), N (4.07,4.00).

Determination of osmium concentrations. Concentrations of Os in test compounds in cell culture medium were quantified using inductively coupled plasma optical emission spectrometry (ICP-OES) for Os using a Perkin Elmer Optima 5300 DV Optical Emission Spectrophotometer. The matrix of calibration standards was matched to the sample matrix by addition of sodium chloride (99.9999% trace metal grade). Data were acquired as instrumental triplicates and processed using WinLab32 V3.4.1 for Windows. Intracellular trace metal concentrations were quantified using inductively coupled plasma mass spectrometry (ICP-MS) for ^{189}Os with an internal standard of ^{166}Er (50 ppb). Data were acquired as instrumental triplicates and processed with MassHunter 3.4 for Windows. In both instances, calibration standards were prepared in 3.6% ultrapure nitric acid supplemented with ascorbic acid (100 mg L^{-1}) and thiourea (10 mM) to stabilise Os in solution and prevent formation of volatile OsO_4.

Biological studies

Cell maintenance. All cell lines used in this study were maintained in pyruvate-free Dulbecco's Modified Eagle Medium (DMEM) supplemented with 10% foetal calf serum, 1% penicillin/streptomycin, and glutamine. All cells were grown as adherent monolayers in a humidified 5% CO_2 environment (37°C) and passaged using trypsin/EDTA (0.25%) upon reaching 90% confluence.

Antiproliferative activity. Briefly, 5 × 10^3 cells were seeded per well (150 µL) in 96-well plates and incubated for 48 h. Separately, stock solutions (typically 100 µM) of test compounds were prepared in culture medium containing dimethylsulfoxide to aid solubility of the osmium catalysts (dimethylsulfoxide working concentrations did not exceed 0.5%) and cells were exposed to six concentrations of test compound (typically 0.1-100 µM, 200 µL) for 24 h. Stock solutions were analysed by ICP-OES to determine the exact concentration of test compound in solution. After 24 h exposure, cells were washed with PBS (200 µL) and incubated for a further 72 h in fresh culture medium (recovery time). Cell viability was determined using the SRB assay. Absorbance measurements were determined using a Thermo Fisher Multiskan FC microplate reader (absorbance at 490 nm). IC_{50} concentrations (viable treated cells compared to untreated controls) were determined as duplicates of triplicates in two independent sets of experiments and standard deviations were calculated. Significance testing was carried out using a two-tailed *t*-test assuming unequal sample variances. Antiproliferative activity experiments were also carried out in the presence of AZD3965 (0, 0.1, 1 or 10 µM) or L-BSO (5 µM) which was administered to cells independently, but within 5 min of administering the metal catalyst.

Cellular accumulation of Os. Briefly, 1 × 10^6 cells were seeded in P100 culture dishes (10 mL) and incubated for 24 h. Media was then removed, and cells were exposed to either: (i) culture medium only, (ii) 10 µM (1 × IC_{50}) compound **1**, (iii) 10 µM (1 × IC_{50}) compound (*R*,*R*)-**1** + 2 mM formate; in culture medium for 24 h. No recovery time was allowed. Cells were harvested using trypsin/EDTA, counted and cell pellets collected for subsequent analysis by ICP-MS. Cell pellets were digested overnight using 200 µL concentrated (72%) trace metal grade nitric acid (80°C) after which time solutions were diluted using doubly-distilled water supplemented with ascorbic acid (100 mg L^{-1}) and thiourea (10 mM) to achieve a final working acid concentration of 3.6% v/v. ^{189}Os quantification was carried out using an Agilent 7900 Series ICP-MS in He-gas collision mode. Experiments were carried out in triplicate with triplicate instrumental replicates of each sample (triplicate of triplicates). Significance testing was carried out using a two-tailed *t*-test assuming unequal sample variances.

Modulation of antiproliferative activity (*in-cell* catalysis). Antiproliferative activities were determined as described above with the following modifications: a fixed (equipotent) concentration of osmium **1** catalyst was used (5.5 µM, 0.5 × IC_{50}), with concentration pre-determined by ICP-OES prior to administration to cells. Sodium formate (0.5, 1.0 or 2.0 mM) was co-administered independently, but within 5 min of addition of catalyst **1**. Cell viability experiments were also carried out using sodium acetate (0-2 mM) in place of sodium formate.

Catalytic reduction of intracellular pyruvate to D-lactate. The catalytic generation of D-lactate was determined as previously described.[17] Briefly, 30 × 10^6 MCF7 human breast cancer cells were seeded in T75 cell culture flasks and incubated for 24 h. After this time, cells were exposed to 11 µM (1 × IC_{50}) of catalyst (*R*,*R*)-**1** or (*S*,*S*)-**1** in combination with 2 mM sodium formate and/or 1 µM AZD3965; in culture medium for 24 h without recovery time. All possible

negative control experiments (osmium catalyst only, sodium formate only, AZD3965 only, sodium formate and AZD3965 only) were also established. Cells were harvested using trypsin/EDTA and cell pellets of equal cell count (40×10^6 cells) were prepared. The D-lactate assay kit (Cayman Chemical) was used to measure intracellular lactate according to the manufactures' instructions. Fluorescence readings were determined using a Promega GloMax Multi+ microplate reader (λ_{ex} 530-540 nm, λ_{em} 585-595 nm). Experiments were carried out in triplicate and standard deviations were reported. Significance testing was carried out using a two-tailed *t*-test assuming unequal sample variances.

Glutathione quantitation. Briefly, 1×10^6 MCF7 human breast cancer cells were seeded in P100 culture dishes (10 mL) and incubated for 48 h. Medium was then removed, and cells were exposed to culture medium containing 11 µM ($1 \times IC_{50}$) compound (*R*,*R*)-**1** either in the presence or absence of AZD3965 (1 µM) and/or sodium formate (2 mM), in culture medium for 24 h without recovery time. All possible negative control experiments (osmium catalyst only, sodium formate and AZD3965 without osmium catalyst) were also established. After this time, cells were washed with PBS (5 mL) and harvested using trypsin/EDTA to obtain cell pellets which were washed with ice cold PBS and lysed by high speed vortexing and centrifugation in combination with freeze/thaw cycling. Glutathione quantitation was achieved using the Glutathione Colorimetric Detection Kit (Invitrogen, purchased from Thermo Fisher Scientific) following the manufacturer's instructions. Experiments were carried out in triplicate and standard deviations are reported. Determined glutathione levels were normalised to cell density, which in turn were converted to molar concentrations by assuming a constant cell volume (1.760×10^{-12} L).[39] Significance testing was carried out using a two-tailed *t*-test assuming unequal sample variances.

This experiment was also carried out with the following modification: cells were treated with 11 µM ($1 \times IC_{50}$) compound (*R*,*R*)-**1** in the presence of 5 µM L-BSO for 24 h without recovery time. Cellular glutathione was quantified as described above.

Table S1. Antiproliferative activities (IC_{50} / µM) for osmium arene catalysts (*R*,*R*)-**1** and **(*S*,*S*)-1** determined in seven human cancer cell lines. 24 h exposure time, 72 h recovery time. Cell viability was determined using the sulforhodamine B assay.

**Cell line**	**Organism**	**Tissue origin**	**IC_{50} / µM**	
			(*R*,*R*)-**1**	(*S*,*S*)-**1**
A2780	*Homo sapiens*	Ovarian	15.5 ± 0.5	15.2 ± 0.5
A549	*Homo sapiens*	Lung	21.1 ± 0.3	20.9 ± 0.4
HCT116	*Homo sapiens*	Colorectal	37 ± 1	36 ± 1
HEPG2	*Homo sapiens*	Liver	29 ± 2	30 ± 4
MCF7	*Homo sapiens*	Breast	10.9 ± 0.7	11 ± 1
OE19	*Homo sapiens*	Oesophageal	> 50	> 50
PC3	*Homo sapiens*	Prostate	12.0 ± 0.3	13.8 ± 0.04

Table S2. Cellular accumulation of osmium (Os fg·cell^{-1}) determined in six human cancer cell lines treated with 1 × IC_{50} concentration of (*R*,*R*)-**1**. 24 h exposure time, no recovery time. Os content determined by ICP-MS analysis of acid-digested cell pellets, normalised to cell count.

**Cell line**	**Organism**	**Tissue origin**	**Os / fg·cell^{-1}**
			(*R*,*R*)-**1**
A2780	*Homo sapiens*	Ovarian	30 ± 2
A549	*Homo sapiens*	Lung	18 ± 2
HCT116	*Homo sapiens*	Colorectal	9.1 ± 0.5
HEPG2	*Homo sapiens*	Liver	17 ± 3
MCF7	*Homo sapiens*	Breast	33 ± 2
OE19 [a]	*Homo sapiens*	Oesophageal	2.22 ± 0.05
PC3	*Homo sapiens*	Prostate	32 ± 2

[a] OE19 cells were treated with 50 µM of Os catalyst 1 (IC_{50} determined > 50 µM)

Table S3. Effect of co-administration of sodium formate with 1 × IC_{50} concentration of catalyst (*R*,*R*)-**1** or (*S*,*S*)-**1** on the accumulation of osmium (Os fg·cell^{-1}) in MCF7 human breast cancer cells treated (n.d. = not determined). 24 h exposure time, no recovery time. Os content determined by ICP-MS analysis of acid-digested cell pellets, normalised to cell count.

	Os / fg·cell^{-1} Sodium formate concentration / mM	
**Catalyst**	**0.0**	**2.0**
(*R*,*R*)-**1**	33 ± 2	33 ± 2
(*S*,*S*)-**1**	32 ± 1	33 ± 3
Untreated	1 ± 1	1 ± 1

Table S4. Antiproliferative activity modulation (normalized cell survival, %) of MCF7 breast cancer cells treated with catalyst (*R*,*R*)-**1** or (*S*,*S*)-**1** in combination with either sodium formate or sodium acetate (0-2 mM). Significance testing (two-tailed *t*-test assuming unequal sample variances) are reported compared to the absence of co-factor administration. 24 h exposure time, 72 h recovery time. Cell viability was determined using the sulforhodamine B assay.* $p < 0.05$, ** $p < 0.01$, *** $p < 0.001$.

		Normalized cell survival / % Cofactor concentration / mM			
**Catalyst**	**Co-factor**	**0**	**0.1**	**1.0**	**2.0**
(*R*,*R*)-**1**	Formate	100 ± 4	85 ± 8 (*p*=0.1009)	77 ± 6 (*p*=0.0117) *	46 ± 4 (*p*=0.0001) ***
(*S*,*S*)-**1**	Formate	100 ± 8	87 ± 8 (*p*=0.1174)	84 ± 5 (*p*=0.0606)	38 ± 4 (*p*=0.0069) **
None	Formate	100 ± 7	94 ± 8 (*p*=0.4004)	100 ± 8 (*p*=1.000)	98 ± 8 (*p*=0.7659)
(*R*,*R*)-**1**	Acetate	100 ± 10	100 ± 7 (*p*=1.0000)	99 ± 8 (*p*=0.9010)	104 ± 9 (*p*=0.6421)
(*S*,*S*)-**1**	Acetate	100 ± 8	89 ± 6 (*p*=0.1528)	94 ± 8 (*p*=0.4103)	93 ± 11 (*p*=0.4384)
None	Acetate	100 ± 8	102 ± 8 (*p*=0.7747)	95 ± 10 (*p*=0.5473)	94 ± 8 (*p*=0.4103)

Table S5. Antiproliferative activity modulation (normalized cell survival, %) of MCF7 breast cancer cells treated with catalyst (*R*,*R*)-**1** in combination with MCT-1 inhibitor AZD3965 (0-10 μM). Significance testing (two-tailed *t*-test assuming unequal sample variances) are reported compared to the absence of co-factor administration. 24 h exposure time, 72 h recovery time. Cell viability was determined using the sulforhodamine B assay. * $p < 0.05$, ** $p < 0.01$.

	Normalized cell survival / % AZD3965 concentration / μM			
**Catalyst**	**0.0**	**0.1**	**1.0**	**10.0**
(*R*,*R*)-**1**	100 ± 5	84 ± 10 (*p*=0.1248)	73 ± 7 (*p*=0.0116) *	71 ± 7 (*p*=0.0099) **
No Os catalyst	100 ± 4	96 ± 4 (*p*=0.2879)	94 ± 5 (*p*=0.2030)	97 ± 11 (*p*=0.7005)

Table S6. Intracellular D-lactate (μM) determined in MCF7 breast cancer cells. Significance testing (two-tailed *t*-test assuming unequal sample variances) are reported compared to the absence of co-factor administration and to the absence of Os catalyst **1**. 24 h exposure time, no recovery time. D-lactate concentration was determined using D-lactate assay kit.

	Intracellular D-lactate / μM Co-factor (none / 2 mM formate / 1 μM AZD3965 / both)			
**Catalyst**	**No co-factor**	**2 mM formate**	**1 μM AZD3965**	**2 mM formate + 1 μM AZD3965**
(*R*,*R*)-**1**	26 ± 3<br>N/A<br>(*p*=0.2646)	52 ± 1<br>(*p*=0.0055) **<br>(*p*=0.0009) ***	27.3 ± 0.5<br>(*p*=0.6835)<br>(*p*=0.0199) *	67 ± 4<br>(*p*=0.0007) ***<br>(*p*=0.0027) **
(*S*,*S*)-**1**	24.3 ± 0.5<br>N/A<br>(*p*=0.3513)	39.2 ± 0.4<br>(*p*=0.0031) **<br>(*p*=0.0001) ***	25.7 ± 0.2<br>(*p*=0.0457) *<br>(*p*=0.9664)	43 ± 3<br>(*p*=0.0073) **<br>(*p*=0.0081) **
No Os catalyst	23.9 ± 0.4<br>N/A<br>N/A	23.5 ± 0.5<br>(*p*=0.3995)<br>N/A	25.7 ± 0.3<br>(*p*=0.0111) *<br>N/A	25.0 ± 0.6<br>(*p*=0.0541)<br>N/A

* $p < 0.05$, ** $p < 0.01$, *** $p < 0.001$. *p*-value relative to co-factor free control; *p*-value relative to the Os catalyst free control

Table S7. Antiproliferative activity modulation (IC_{50}, μM) of MCF7 breast cancer cells treated with catalyst (*R*,*R*)-**1** in the presence or absence of AZD3965 (1 μM). 24 h exposure time, 72 h recovery time. Cell viability was determined using the sulforhodamine B assay.

	IC_{50} concentration / μM	
**Catalyst**	**No co-administration**	**1 μM AZD3965**
(*R*,*R*)-**1**	11 ± 1	8 ± 2

Table S8. Antiproliferative activity modulation (IC_{50}, μM) of MCF7 breast cancer cells treated with catalyst (*R*,*R*)-**1** in the presence or absence of L-BSO (5 μM). Significance testing (two-tailed *t*-test assuming unequal sample variances) are reported compared to the absence of co-factor administration. 24 h exposure time, 72 h recovery time. Cell viability was determined using the sulforhodamine B assay.

	IC_{50} concentration / μM	
**Catalyst**	**No co-administration**	**5 μM L-BSO**
(*R*,*R*)-**1**	10.9 ± 0.7	8.9 ± 0.8 (*p*=0.0472) *

* $p < 0.05$, ** $p < 0.01$, *** $p < 0.001$.

Table S9. Determination of total intracellular glutathione concentration (mM) in MCF7 breast cancer cells treated with (*R*,*R*)-**1** in combination with a co-factor (none / 2 mM formate / 1 μM AZD3965 / both). Significance testing (two-tailed *t*-test assuming unequal sample variances) are reported compared to the absence of co-factor administration. 24 h exposure time, no recovery time. Cell viability was determined using the glutathione assay kit (Thermo Scientific).

	Intracellular glutathione / mM<br>Co-factor (none / 2 mM formate / 1 μM AZD3965 / both)<br>*P-value relative to co-factor free control*			
**Catalyst**	**No co-factor**	**2 mM formate**	**1 μM AZD3965**	**2 mM formate + 1 μM AZD3965**
(*R*,*R*)-**1**	3.5 ± 0.7	3.4 ± 0.3 (*p*=0.8412)	2.0 ± 0.4 (*p*=0.0485) *	1.7 ± 0.6 (*p*=0.0430) *
No Os catalyst	3.9 ± 0.4	3.6 ± 0.3 (*p*=0.3751)	2.4 ± 0.5 (*p*=0.0270) *	2.5 ± 0.2 (*p*=0.0324) *

* $p < 0.05$, ** $p < 0.01$, *** $p < 0.001$.

Table S10. Determination of total intracellular glutathione concentration (mM) in MCF7 breast cancer cells treated with (*R*,*R*)-**1** in the presence of 5 µM L-BSO, an inhibitor of glutathione synthesis. Significance testing (two-tailed *t*-test assuming unequal sample variances) is compared to the absence of co-factor administration. 24 h exposure time, no recovery time. Cell viability was determined using the glutathione assay kit (Thermo Scientific).

	Intracellular glutathione / mM<br>Co-factor (none / 5 µM L-BSO)	
**Catalyst**	**No co-factor**	**5 µM L-BSO**
(*R*,*R*)-1	3.5 ± 0.7	1.1 ± 0.4<br>(*p*=0.0141) *
No Os catalyst	3.9 ± 0.4	1.1 ± 0.8<br>(*p*=0.0324) *

* $p < 0.05$, ** $p < 0.01$, *** $p < 0.001$; *p-value relative to co-factor free control*
